# A Systematic Review and Meta-Analysis of the Clinical Appropriateness of Blood Transfusion in China

**DOI:** 10.1097/MD.0000000000002164

**Published:** 2015-12-18

**Authors:** Changtai Zhu, Yulu Gao, Zhiqiang Li, Qinyun Li, Zongshuai Gao, Yanqiu Liao, Zhifeng Deng

**Affiliations:** From the Department of Transfusion Medicine, Shanghai Jiao Tong University Affiliated Sixth People's Hospital, Shanghai (CZ, ZL, QL, ZG), Department of Laboratory Medicine, Kunshan Hospital Affiliated to Nanjing University of Traditional Chinese Medicine, Kunshan, Jiangsu Province (YG), Department of Neurosurgery, Shanghai Jiao Tong University Affiliated Sixth People's Hospital, Shanghai (ZD), and Department of Transfusion Medicine, Anhui Provincial Hospital, Anhui Medical University, Hefei, China (YL).

## Abstract

The issue of the clinical appropriateness of blood transfusion has become a focus of transfusion medicine worldwide. In China, irrational uses of blood have often been reported in recent years. However, to date there lacks a systematic review of the rational uses of blood.

This study aimed to determine the clinical appropriateness of blood transfusion in China.

We searched PubMed, Web of Science, the Cochrane Library, China National Knowledge Infrastructure (CNKI), China Science and Technology Journal Database, WanFang Database, and Chinese BioMedical Literature Database, and the retrieval cut-off date was June 31, 2015. SPSS 17.0 and MetaAnalyst 3.13 were employed as the statistics tools in this review. A pooled rate of clinical inappropriateness of transfusion was analyzed by DerSimonian–Laird method.

In this study, a total of 39 observational studies were included, which related to 75,132 cases of blood transfusion. According to the meta-analysis results, the overall incidence of clinical inappropriateness of transfusion in China was estimated to be 37.3% (95% confidence interval [CI] [32.1, 42.8]). The subgroup analyses revealed that the pooled rates of clinical inappropriateness of transfusion of plasma, red blood cells (RBCs), cryoprecipitate, and platelets were 56.3% (95% CI [45.8, 66.2]), 30.9% (95% CI [27.1, 35.0]), 25.2% (95% CI [13.2, 42.7]), and 14.1% (95% CI [8.8, 21.9]), respectively. However, the pooled incidence of inappropriateness of transfusion in operative departments was 47.5% (95% CI [36.8, 58.3]), which was significantly higher than that in nonoperative departments, 25.8% (95% CI [18.7, 34.4], *P* < 0.05). The overall rates of inappropriate use were 36.7% (95% CI [30.2, 43.6]) in major cities and 37.5% (95% CI [31.2, 44.3]) in other cities, respectively; there was no statistically significant difference (*P* > 0.05).

In conclusion, China has suffered from a disadvantage in the clinical appropriateness of blood transfusion, especially in plasma and RBC use. In future, comprehensive measures should be implemented in order to improve the clinical appropriateness of blood transfusion.

## Abstract 2

Supplemental Digital Content is available in the text

## INTRODUCTION

Blood transfusion has become an irreplaceable means of clinical treatment. However, transfusion related adverse events, such as immunologic reaction and infection, have been reported.^1–11^ Previous studies have shown restrictive transfusion strategies are superior to liberal transfusion strategies.^12–20^ According to a systematic review and meta-analysis published in 2015, compared with liberal strategies, restrictive transfusion strategies were associated with a reduction in the number of red blood cell (RBC) units transfused and number of patients being transfused, but disadvantageous outcomes seemed to be unaltered.^19^ Restrictive transfusion strategies are safe in most clinical settings, and liberal transfusion strategies have not been shown to convey any benefit to patients.^19^ However, another recent meta-analysis showed that, in patients with critical illness or bleed, restricting blood transfusions by using a hemoglobin trigger of <7 g/dL significantly reduces cardiac events, rebleeding, bacterial infections, and total mortality.^20^ The World Health Organization proposed the rational use of blood and blood products to reduce unnecessary transfusions and minimize the risks associated with transfusion.^21^ Many countries have developed national guidelines on the appropriate clinical use of blood.^22–32^ The Ministry of Health of the People's Republic of China formulated guidelines for blood transfusion in 2000^33^ and the regulations for clinical management of blood use in medical institutions in 1999 and 2012.^34,35^ Moreover, in 2010, a national program for improvement of medical quality was launched by the Ministry of Health, which especially emphasized the rational use of blood and the conduct of relevant audits.^36^ In response, the problem of appropriate blood use has caused widespread concern among the Chinese, and irrational uses of blood have often been reported in recent years. However, considering the lack of a systematic review and meta-analysis of rational uses of blood in China, we performed the current study to clarify the relevant issues.

## MATERIALS AND METHODS

### Ethnic Statement

All analyses in this study were based on the previous published papers. Therefore, ethical approval and patient consent are not required.

### Eligibility Criteria

The inclusion criteria were as follows: observational studies that reported the incidence of inappropriateness of blood transfusion; studies that used the Specifications and Guidelines of Clinical Blood Transfusion Technology^33^ as the standard to determine blood transfusion appropriateness; studies carried out in China; studies involved in clinical blood transfusion cases; studies that reported the total number of cases of blood use and the cases of blood inappropriate use; and design type was observational study. Exclusion criteria were as follows: the records were not related to this study; incorrect or inconsistent data were confirmed; and the records were confirmed to be repeated or indexed in different databases.

### Information Sources

The following electronic databases were searched: PubMed, Web of Science, the Cochrane Library, China National Knowledge Infrastructure (CNKI), China Science and Technology Journal Database, WanFang Database, and BioMedical Literature Chinese Database. Additional data and information were solicited from the authors of the included studies.

### Search Strategy for Identification of Studies

The retrieval terms used were “blood transfusion,” “blood use,” “clinical,” “appropriateness,” “inappropriateness,” “China,” “blood product,” “blood component,” “irrational,” “irrationality” “rational,” “plasma,” “red blood cell,” “white blood cell,” “cryoprecipitate,” “granulocyte,” and related entry terms. The terms were searched in different combinations. Limits for language were not used in this study. The retrieval cut-off time was June 31, 2015.

### Study Selection, and Quality and Bias of Studies

In the study, 2 independent reviewers (QYL and ZSG) independently screened the references in accordance with the inclusion and exclusion criteria and extracted the data according to a format table. Disagreements were resolved by consensus or by a third reviewer (CTZ).

Loney scoring system^37^ was employed as an analytic tool for assessing the methodological quality in this study. The Loney scoring system provides a checklist including 8 items (each item was assigned a score of 1 point, totaling 8 points): random sample or whole population, unbiased sampling frame, adequate sample size, standardized measures, outcomes measured by unbiased assessors, adequate response rate (at least 70%), and description of refusers, confidence interval (CI) and subgroup analysis, and all study subjects described.^37,38^ Scores of 0 to 4 indicated lower quality, while scores greater than 5 suggested higher quality.^38^

### Statistical Analysis

Statistical calculations were performed using the Statistical Package for Social Sciences program (SPSS, Chicago, IL) version 17.0 and MetaAnalyst version 3.13 (Boston, MA), and the pooled rate with 95% CI was automatically calculated. The heterogeneity test was performed by homogeneity test. Heterogeneity among the studies was evaluated by the Cochran Chi-squared test (Cochran Q) and the I^2^ statistics. If there was heterogeneity among the included studies, a random-effect model (REM) to pool the incidences was used; otherwise, a fixed-effect model (FEM) was selected. In this study, the DerSimonian–Laird method was used for the REM.^39^ In addition, Begg funnel plot by MetaAnalyst version 3.13 was used for the observation of the potential publication bias.

## RESULTS

### Study Selection

**FIGURE 1:**
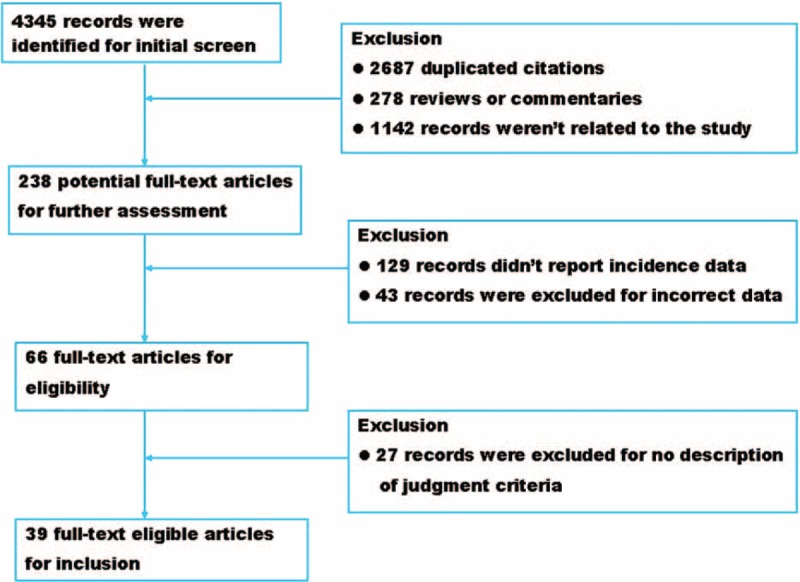
The flow chart of database retrieval and literature screening in this study.

A total of 4345 records were retrieved from the databases, 66 of which were selected for full-text assessment for eligibility. Finally, 39 observational studies involving 75,132 transfusion cases were included in this study.^40–78^ The process of database retrieval and literature screening is shown in Figure 1. The review protocols were in accordance with the Preferred Report Items for Systematic Reviews and Meta-analyses 2009 Checklist (Supplemental file 1).

### Characteristics and Quality Evaluation of Included Studies

The studies included 75,132 cases of blood transfusion, performed in 44 cities under the jurisdiction of 17 provinces and 2 municipalities in China. As far as the geographical and population distribution were concerned, the sampling in this study was representative. In the study, the sample size ranged from 61 to 21,687 cases, a median of 1060; however, among subgroups, the sample size had great difference (Table 1). The Loney system score evaluation revealed that the range of the report quality scores was from 5 to 6 points and that the average quality score was 5.6 points. The overall quality of the literature was high. The characteristics and quality evaluation of included studies is shown in Table 2.

**TABLE 1:**
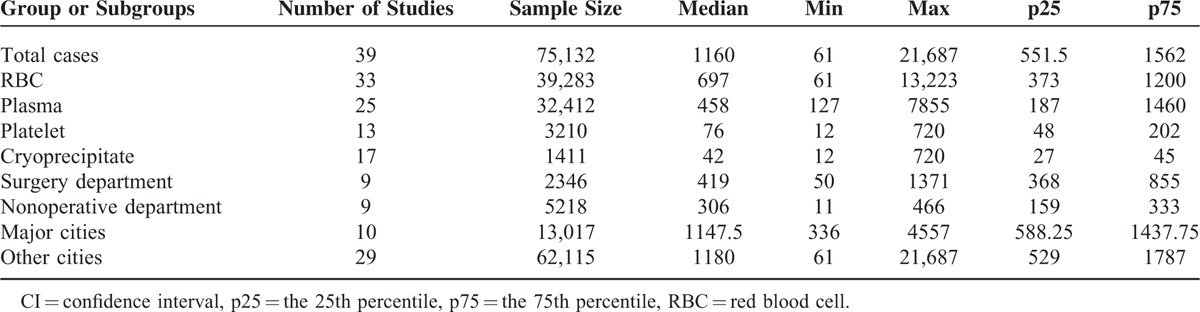
Sample Analysis in Each of Groups in This Study

**TABLE 2:**
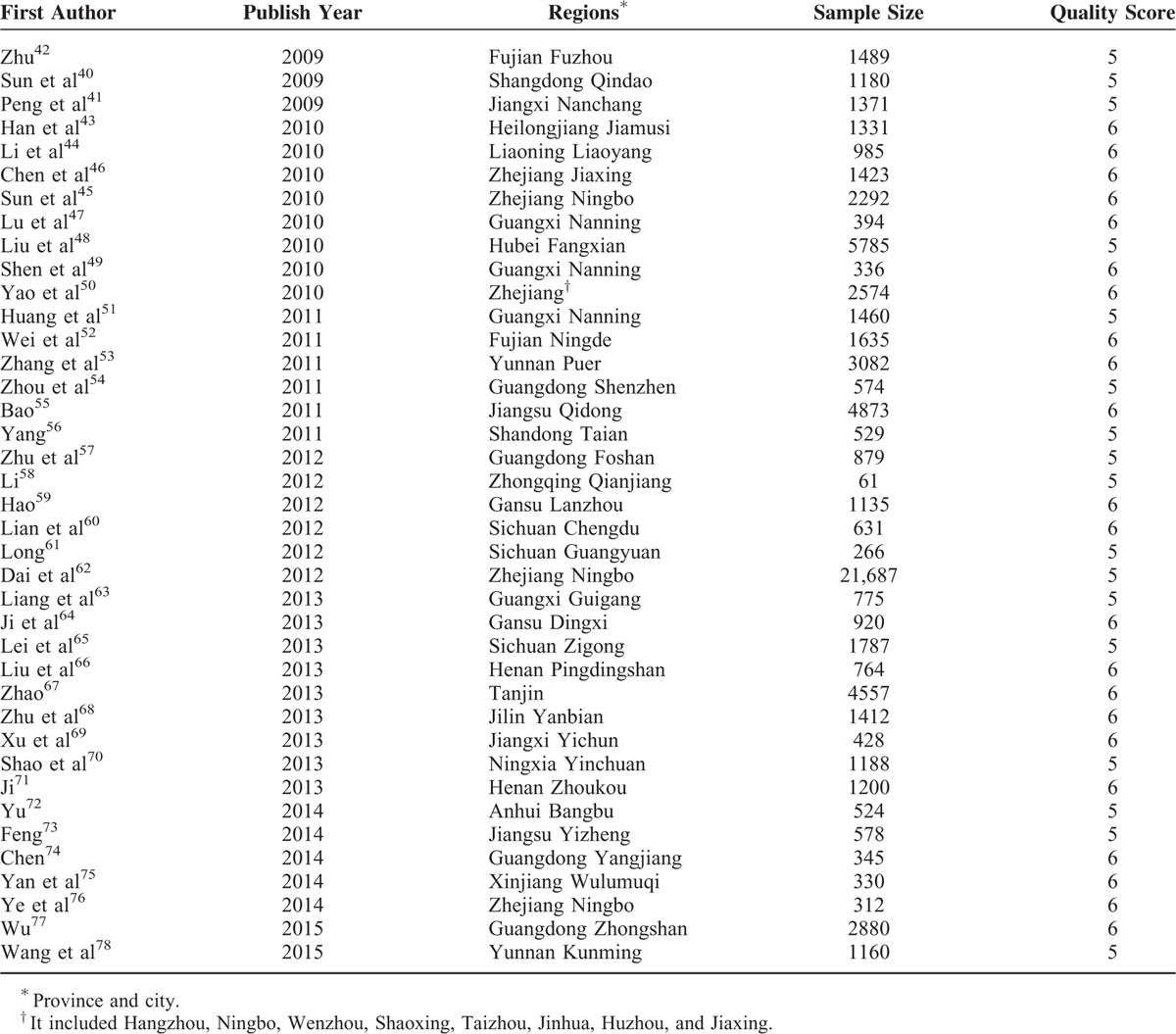
General Characteristics and Quality Evaluation of the Included Studies

### Synthesis of Results

**FIGURE 2:**
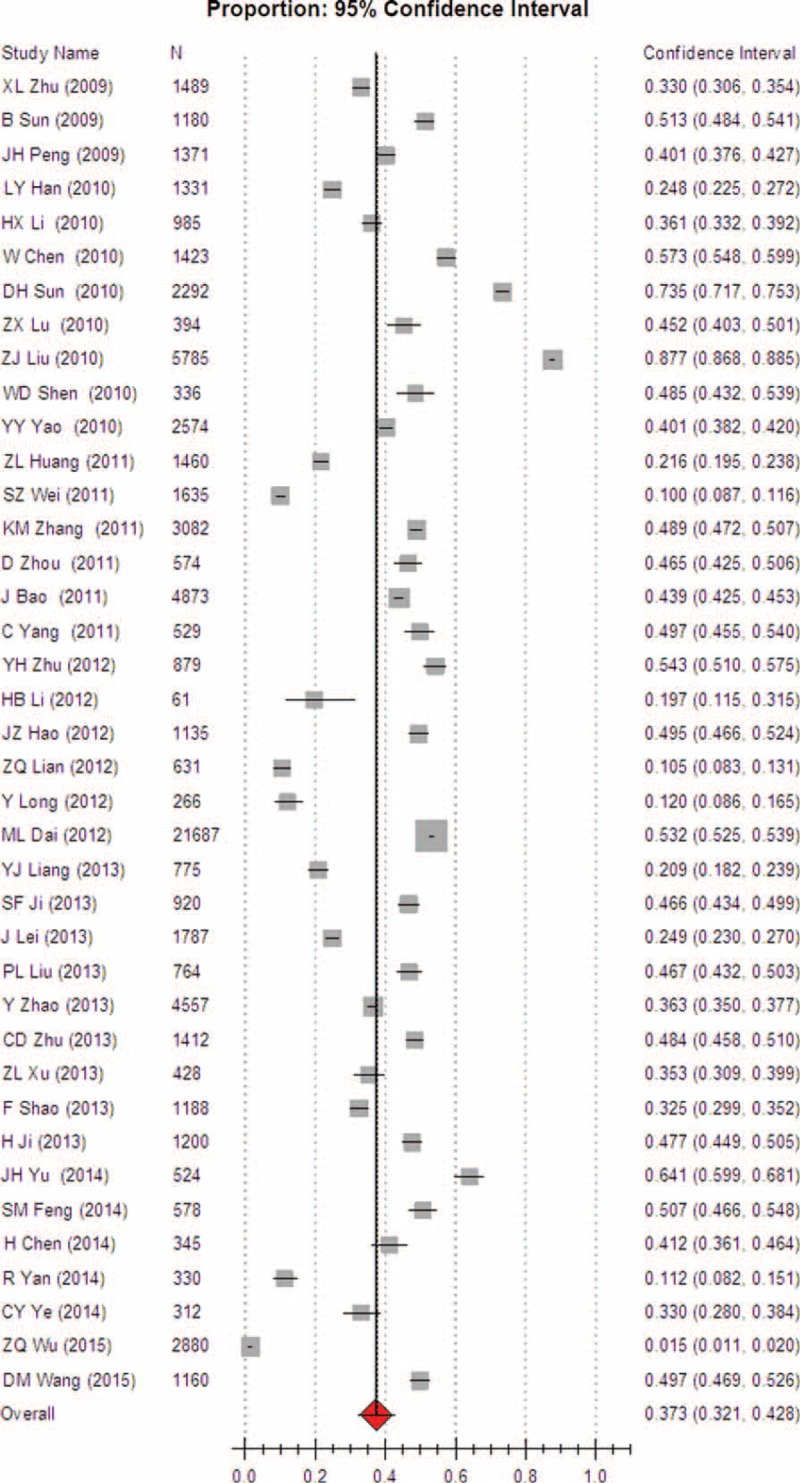
Meta-analysis of the overall rate of clinical irrational blood use.

**FIGURE 3:**
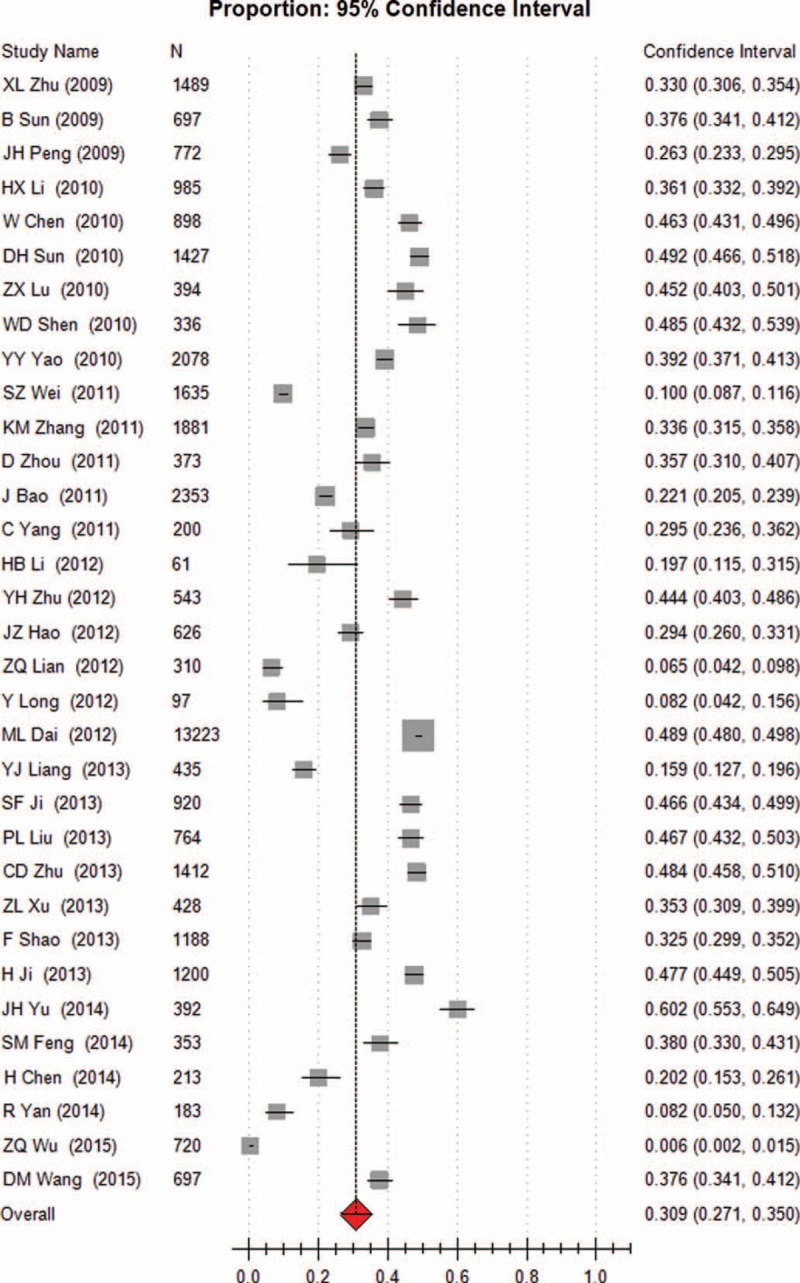
Meta-analysis of the pooled rate of clinical irrational use in red blood cell.

**FIGURE 4:**
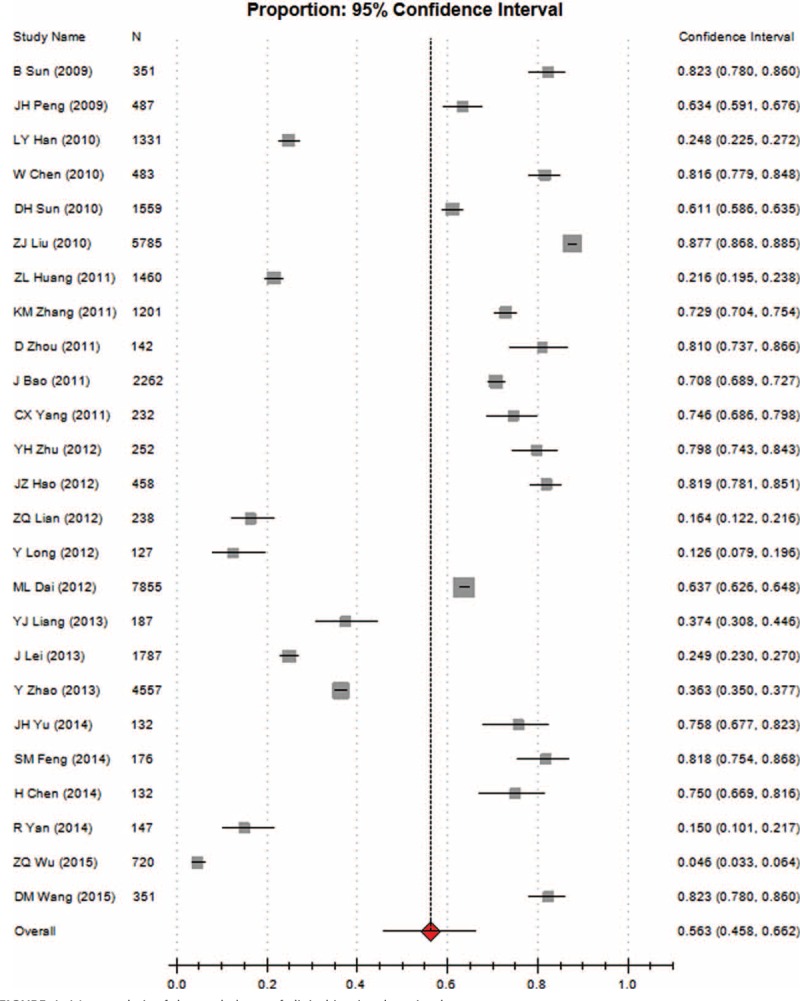
Meta-analysis of the pooled rate of clinical irrational use in plasma.

**FIGURE 5:**
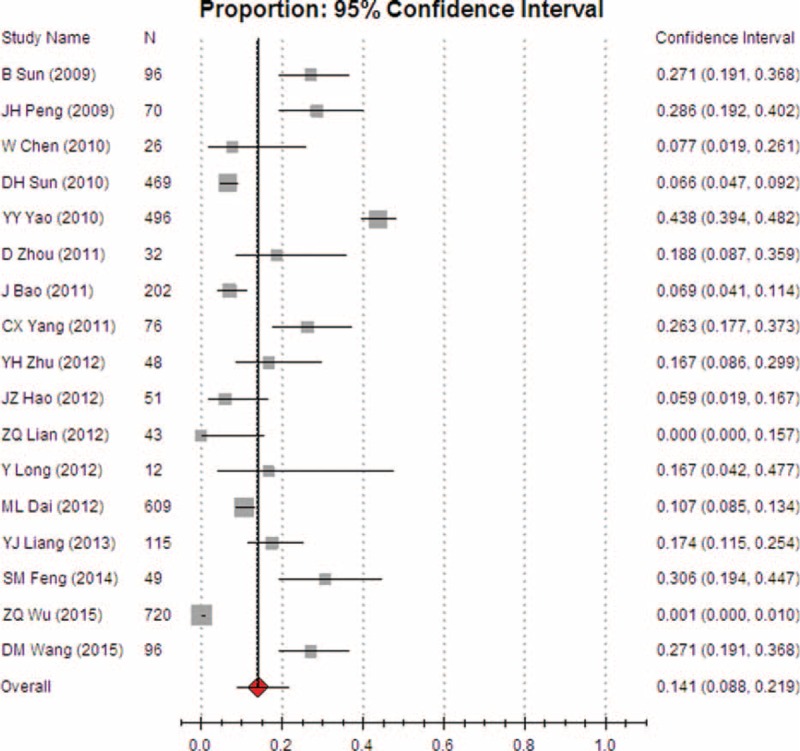
Meta-analysis of the pooled rate of clinical irrational use in platelet.

**FIGURE 6:**
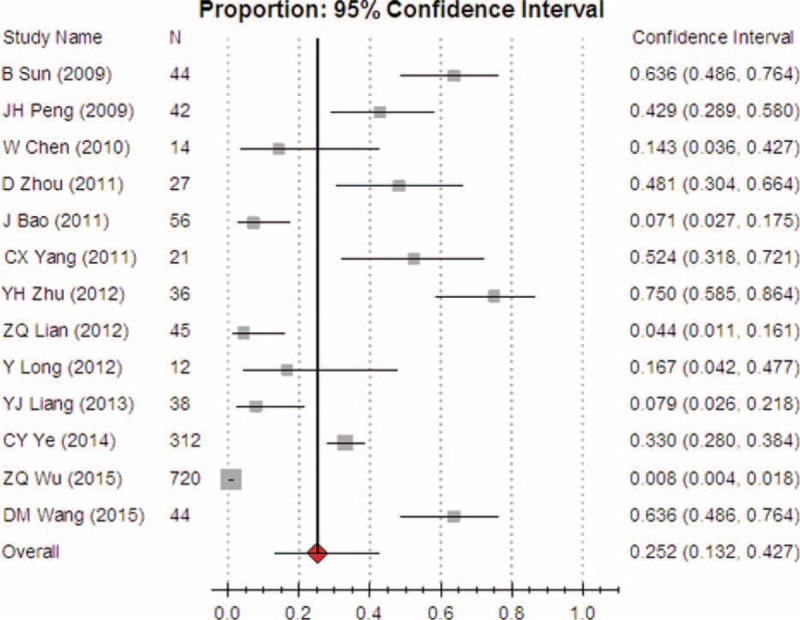
Meta-analysis of the pooled rate of clinical irrational use in cryoprecipitate.

Heterogeneity tests and the main results of the meta-analysis are shown in Table 3. The results of the meta-analysis indicated that the overall rate of clinical irrational blood use was 37.3% (95% CI [32.1, 42.8], REM) (Fig. 2). Further subgroup analysis showed that the pooled rate of clinical irrational transfusion in RBC use was 30.9% (95% CI [27.1, 35.0], REM) (Fig. 3); in plasma use it was 56.3% (95% CI [45.8, 66.2], REM) (Fig. 4); in platelet use it was 14.1% (95% CI [8.8, 21.9], REM) (Fig. 5); and in cryoprecipitate use it was 25.2% (95% CI [13.2, 42.7], REM) (Fig. 6).

**TABLE 3:**
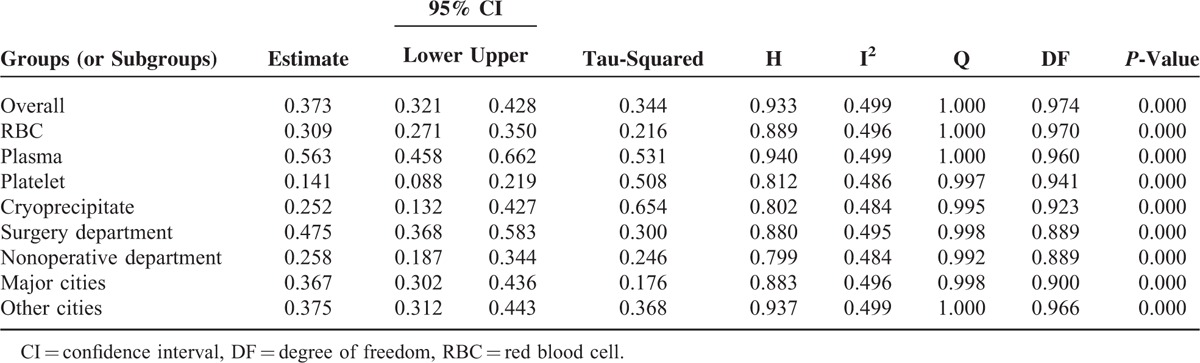
Heterogeneity Tests of Meta-Analysis in This Study

**FIGURE 7:**
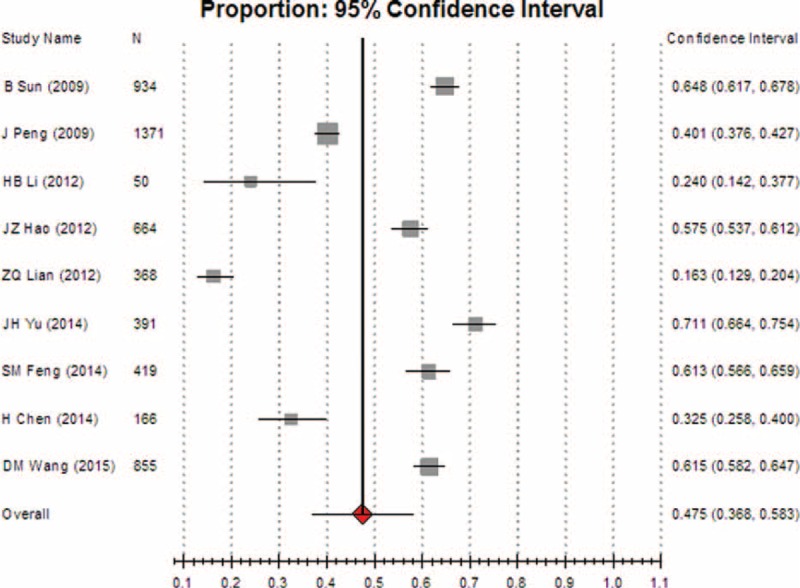
Meta-analysis of the pooled rate of clinical irrational use in operative departments.

**FIGURE 8:**
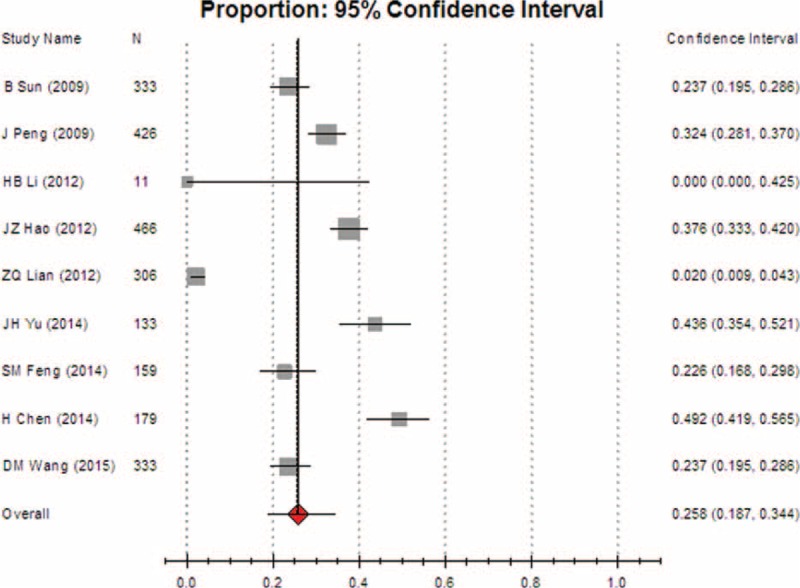
Meta-analysis of the pooled rate of clinical irrational use in nonoperative departments.

Comparisons of subgroups showed that the pooled incidence of inappropriate transfusion in surgery departments was 47.5% (95% CI [36.8, 58.3], REM) (Fig. 7), while the incidence in nonoperative departments was 25.8% (95% CI [18.7, 34.4], REM) (Fig. 8). The pooled rate in surgery departments was significantly higher than that in nonoperative departments (*P* < 0.05).

**FIGURE 9:**
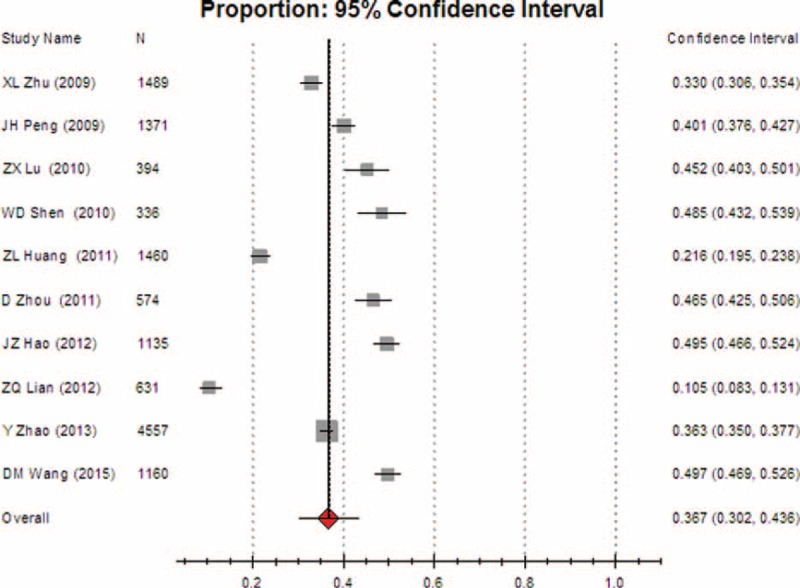
Meta-analysis of the pooled rate of clinical irrational use in major cities.

**FIGURE 10:**
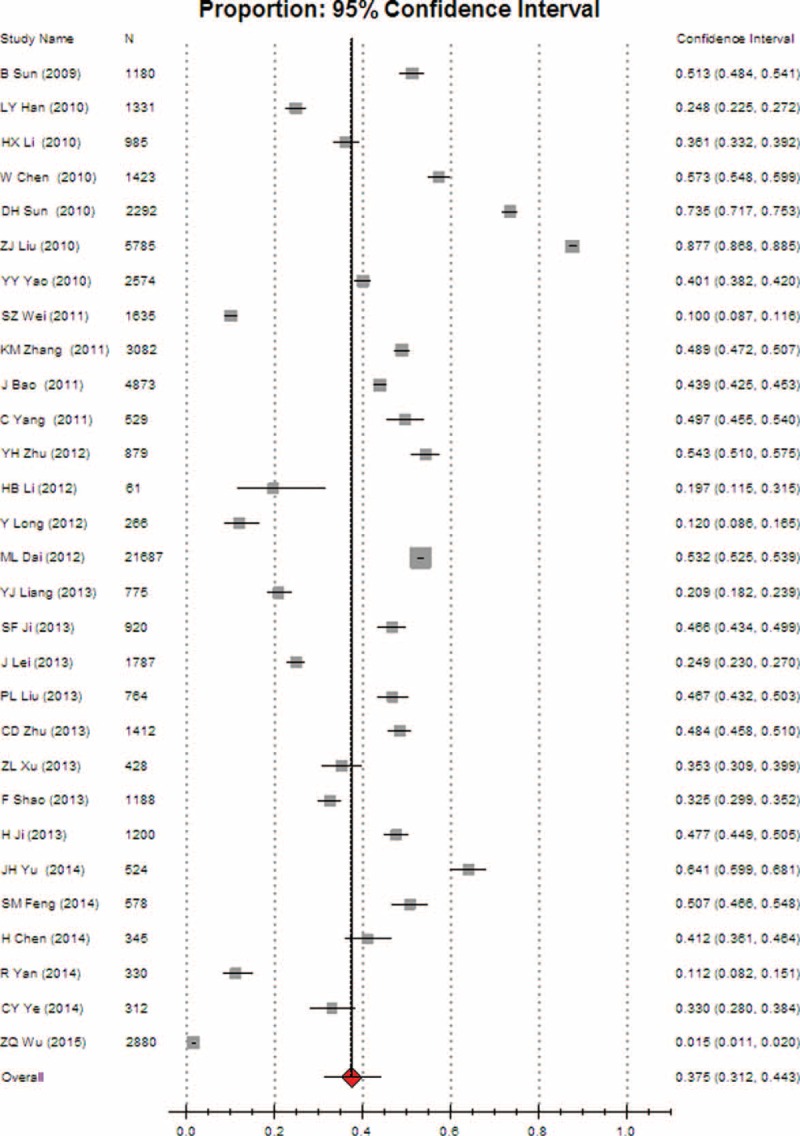
Meta-analysis of the pooled rate of clinical irrational use in other cities.

The overall rates of inappropriate use were 36.7% (95% CI [30.2, 43.6], REM) in major cities (including municipalities and provincial capitals) (Fig. 9) and 37.5% (95% CI [31.2, 44.3], REM) in other cities (Fig. 10), respectively. No statistically significant difference was found between them (*P* > 0.05).

### Publication Bias

**FIGURE 11:**
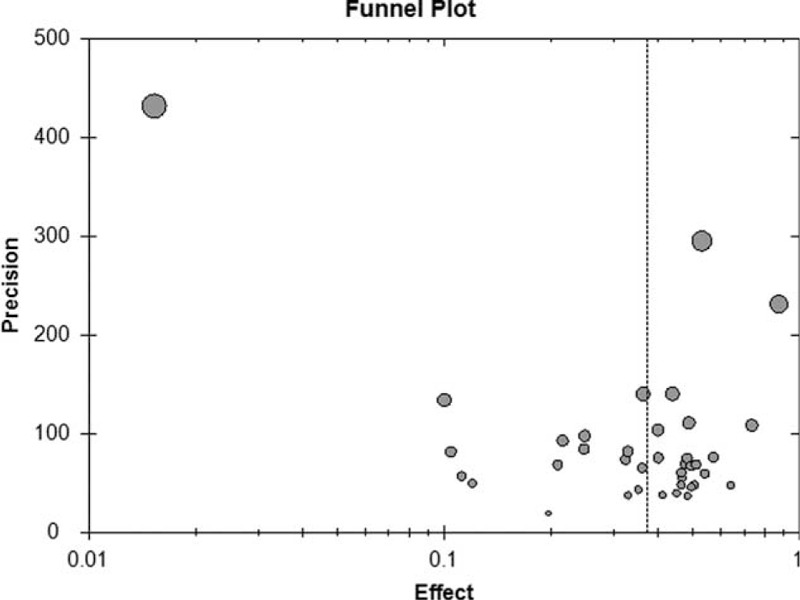
Funnel plot for the observation of the publication bias.

The funnel plot was asymmetric (Fig. 11), suggesting that a publication bias occurred in this study.

## DISCUSSION

Blood and blood products are a limited resource and therefore should be used effectively. The irrational use of blood is not only a waste of precious medical resources, but also increases the blood transfusion risk and economic burden. Therefore, research into the rationality of clinical blood transfusion and subsequent management is warranted. In recent years, the issue of the appropriateness of blood use has become a focus.^79–93^ In 2012, 70% of countries had a national blood policy. Overall, 62% countries (81% high-income countries, 60% middle-income countries, and 44% low-income countries) had specific legislation covering the management of blood transfusion.^20^ Currently, most developed countries have implemented a national guideline for blood use. However, some studies have reported that there are still different degrees of inappropriate blood use in these countries.^84–93^

According to a previous report from Sydney, New South Wales, and Australia, a total of 1117 medical records from 10 major urban hospitals in 1998 and 1999 were audited in order to assess the appropriateness of RBC transfusions. The authors of the report found that about a 3rd of RBC transfusions were assessed as inappropriate, and more RBC transfusions were inappropriate in surgical patients than in those treated by other specialties.^92^

A recent study in a tertiary hospital in the United States reported that the implementation by blood bank staff of more rigorous prospective audits of orders for blood products resulted in a significant decrease of 9.1% and 10.3% in the use of fresh frozen plasma and platelets, respectively.^93^ In China, although most urban hospitals have established a Hospital Transfusion Committee as required by the relevant regulations since 2009, the current status of appropriate blood use seems to not be satisfactory, based on the published reports in recent years.

The above facts show that both developed and developing countries have issues with appropriate blood use. More attention should be paid to this issue worldwide. However, to date, there has not been a systematic review and meta-analysis of irrational uses of blood in China or in other countries. Therefore, we performed this study to clarify the relevant issues.

In this study, we included 39 reports involving 75,132 cases of blood transfusion in China. As far as geographical and population distribution was concerned, the sampling in this study was representative. In this study, the Loney system score evaluation revealed that the report quality ranged from 5 to 6 points and that the average quality score was 5.6 points, suggesting that the overall quality of the included studies was high.

Meta-analysis results revealed that the overall rate of clinical irrational blood use was 37.3% (95% CI [32.1, 42.8]), indicating that a considerable proportion of blood products have been irrationally used in China in recent years.

Further subgroup analysis showed that the pooled rates of clinical irrational transfusion of plasma, RBC, cryoprecipitate, and platelet were 56.3% (95% CI [45.8, 66.2]), 30.9% (95% CI [27.1, 35.0]), 25.2% (95% CI [13.2, 42.7]), and 14.1% (95% CI [8.8, 21.9]), respectively. These results suggested that among each of the blood components that we observed, the overall appropriateness was worrying.

According to the meta-analysis results, clinical appropriateness was the lowest for plasma transfusion, followed by RBC, cryoprecipitate, and platelet transfusion. The causes of the difference were complicated; however, we found that one of the most important factors may be linked to the supply shortage of these blood components. In China, the shortage of platelet supplies is most serious, followed by cryoprecipitate and RBC, while shortages of plasma supplies are relatively mild. We believe that the extent of the supply shortage of blood products plays an important role in determining the clinical practice of physicians, influencing the implementation of policies and management measures related to blood product use. For example, the pretransfusion audits of platelets are more rigorous than plasma use in clinical practice.

In reviewing the literature, we found that the main phenomena for irrational use of clinical blood were as follows: blood transfusion indications were incorrect; and the volume of blood transfusion was incorrect (beyond the limits recommended by the guidelines).^32^ Furthermore, we generalized that the main causes of irrational clinical blood use were: lack of transfusion knowledge amongst clinicians; low efficiency with respect to blood transfusion management; blood substitutes not available (the shortage of some plasma-derived medicinal products has often occurred in China); and unnecessary blood transfusion due to the worry of death of patients after operation. However, we also noted that the main reasons for irrational plasma use by clinicians were as follows: to improve immunity; to add up to nutrition; to increase circulating blood volume; and to supply blood albumin (plasma is more easily available and is cheaper compared to commercial albumin in China). Considering that the issue of irrational use was the most prominent for plasma, more rigorous measures should be taken to constrain and regulate the clinical use of plasma in the future.

We also found that the pooled rate of inappropriateness transfusion in surgery departments was 47.5% (95% CI [36.8, 58.3]), which was significantly higher than that in nonoperative departments, suggesting that a large difference in irrational use exists among different departments. Hence, more attention should be paid in the future to the problem of irrational transfusion in surgical patients.

Through reviewing the literature, we found that differences in the appropriateness of blood use in the different regions were closely associated with the implementation of the relevant regulations, the transfusion indicator audits, and the physicians’ compliance with the guidelines on blood transfusion. However, subgroup comparison revealed that there was no statistically significant difference in the overall rates of inappropriate use between major cities and other regions, suggesting that China has suffered from universal irrational blood uses.

None of the retrieved studies reported data for granulocyte transfusion and therefore, we did not assess the appropriateness of granulocyte transfusion. In addition, we also noted that whole blood had been rarely used and blood component transfusion had become a routine in clinical practice in recent years through the review of the literatures.

The limitations of this study were as follows:

- The asymmetric funnel plot suggests that publication bias is affecting this study, indicating that studies with larger effects were more likely to be published and therefore included in the current review. In addition, the gray literature such as dissertations and unpublished data were not included in the current meta-analysis, which might exaggerate the effect sizes of the meta-analysis results.
- Some studies did not report whether outcomes were measured by unbiased (blinded) assessors.
- There was large variability in sample size and the incidence of inappropriate use among different studies, which conferred heterogeneity to this study.
- The lack of data in some provinces might have affected the results.

Therefore, more powerful evidence and more scientific research are needed in future to clarify the rational use of blood.

At present, under the influence of cultural values and other factors, the rate of voluntary blood donation in the Chinese population is lower compared with developed countries, which has resulted in a shortage of clinical blood supply, and even in delayed operations. However, according to the above results, a considerable proportion of blood components were irrationally used, suggesting that some of the available blood resource in China was potentially wasted. Therefore, it is important that more effective measures are taken to improve the management of blood use in China. We recommend that the following methods be adopted in the future: implementation of mandatory pretransfusion approval programs such as using a computer as a guide to pretransfusion evaluation;^82,94^ improving the blood transfusion skills and evidence-based medicine knowledge of clinicians;^80,95–99^ conferring pretransfusion audit to transfusion appropriateness for blood bank staff or hematologists;^100,101^ and establishing more effective blood transfusion management systems, such as a national internet platform for monitoring the rational use of blood. In addition, we introduced an automatically computerized calculator for assessing the precise transfusion amount of RBC, plasma, and cryoprecipitate and the effectiveness of platelet transfusion (Supplemental file 2) in this study. By the useful software, the complicated calculation process for the pretransfusion evaluation becomes very simple; therefore, we recommend the original application software to the clinicians for the pretransfusion evaluation in clinical practice. We believe that introduction of these comprehensive measures will greatly improve the rational use of blood in China.

In conclusion, China has suffered from inappropriate clinical use of blood transfusions, especially plasma and RBC use. In future, comprehensive measures should be implemented to improve the clinical appropriateness of blood transfusion.

## Supplementary Material

Supplemental Digital Content
